# Fibrous dysplasia – differential diagnosis of cystic lesions in the proximal femur:a case report

**DOI:** 10.1186/1757-1626-2-26

**Published:** 2009-01-08

**Authors:** Stefan Endres, Axel Wilke

**Affiliations:** 1 Department of orthopaedic surgery Elisabeth-Klinik GmbH Bigge/Olsberg, Heinrich-Sommer-Str. 4, 59939 Olsberg, Germany

## Abstract

**Background:**

We are reporting about the case of a 22-year old woman, who made a first visit as an outpatient with pain that arose in acute form in the area of the left groin. The patient history did not indicate a preceding trauma, or an inflammatory or malignant disease.

**Case presentation:**

Conventional X-ray showed the image of a cystic lesion in the area of the neck of the thigh [collum femoris] with pathological fracture in the area of the calcar. The MRT produced the diagnosis of an aneurismal bone cyst, in which the presence of juvenile bone cyst or a giant cell tumor could be taken into consideration by differential diagnosis.

Owing to the pathological fracture, repair by operation of the lesion was carried out by means of spongioplastic surgery and internal fixation (DHS).

**Conclusion:**

Histology produced characteristic findings fitting the condition of fibrous dysplasia, which was not included among the primary causes in the considerations offered by differential diagnosis.

Therefore, this case is a good example of the fact that fibrous dysplasia must be taken into account when using differential diagnosis as one of the cystic lesions.

## Background

Polyostotic fibrous dysplasia is a rare disease, which was already described in 1938 by Lichtenstein. In a later publication with Jaffé he also described the monostotic form of the disease [1]. Today we divide fibrous dysplasia into several forms and syndromes: the monostotic form, the polyostotic form, the McCune-Albright-Syndrome and the Mazabraud-Syndrome.

Owing to the different manifestation forms of the symptoms, when making the diagnosis of the polyostotic form as well as of McCune-Albright-Syndrome, most cases already occur in young age (85% between the 2^nd ^and 30^th ^year of life), whereas the monostotic form is often detected as an accidental finding. Fibrous dysplasia represents approximately 2,5% of all bone lesions, as well as 7% of all benign bone tumors [2]. The distribution shows minimal preference towards the female sex (m:f = 1:1,2) [3]. However, present international epidemiological data are not available.

## Case presentation

We are reporting about the case of a 22-year old woman, who made a first visit as an outpatient with pain that arose in acute form in the area of the left groin. The complaints occurred in acute form without preceding trauma and led to inability to put stress on the left leg. Until the occurrence of this acute pain the patient was free of complaints and active in sports.

The physical examination produced pain on pressure in the groin as well as pronounced pain on movement of the left hip joint mainly during inner rotation. There were no neurological deficits. Soft tissue, blood circulation and sensorimotor function of the lower extremities were intact. Similarly there were no signs of an acute infection or general disease symptoms such as fever, night sweat or loss of weight. The ultrasound of the inguinal region carried out subsequently revealed an intraarticular exudate of the left hip joint, so that conventional X-ray examination was initiated.

**Figure 1:**
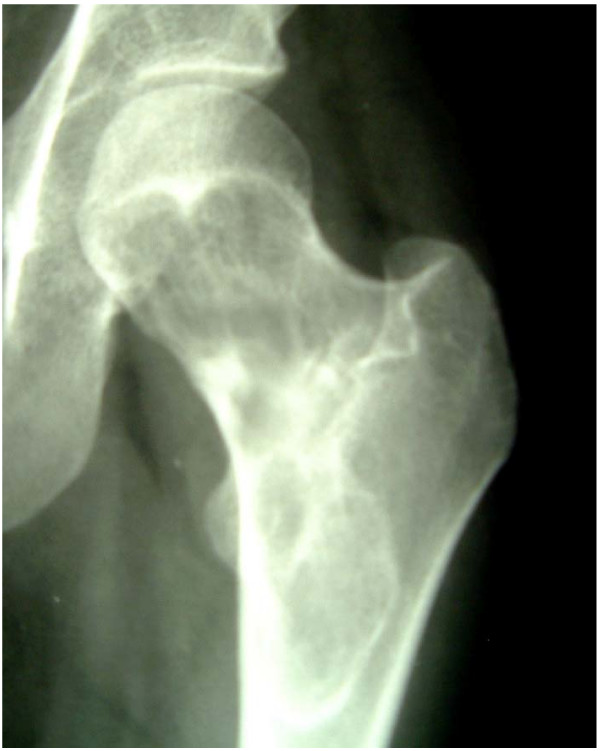
**X-Ray, preoperative: Cystic lesion of the proximal femur**.

In the overview of the pelvis and of the axial image of the left hip joint an osteolytic lesion appeared in the area of the proximal femur with partially compartmented portions and reactive peripheral sclerosis. In addition a pathological fracture was remarkable in the area of the calcar, which explained the complaints of the patient. (Fig. 1)

**Figure 2:**
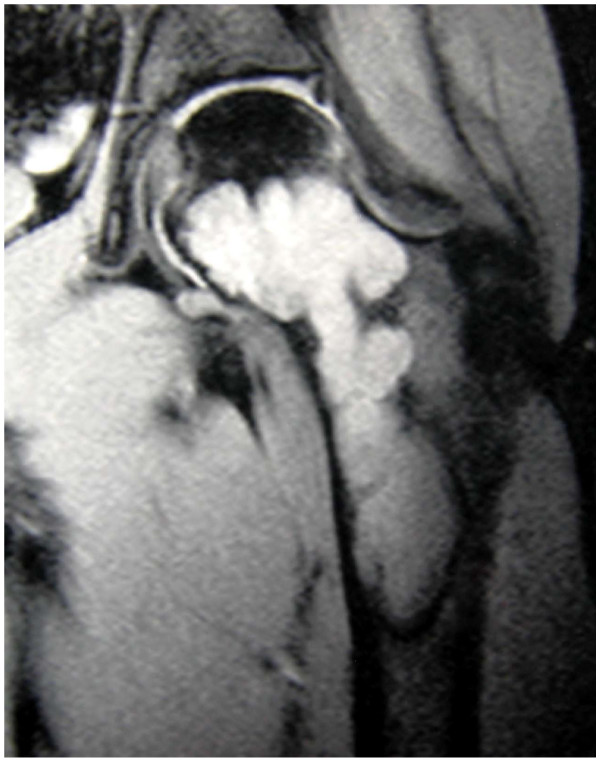
**MRI, preoperative: Cystic lesion of the proximal femur**.

An MRI of the pelvis produced a large, cystic space occupying lesion of the neck of thigh [collum femoris] and proximal femur without sign of malignant degeneration. (Fig. 2)

The blood test showed an increase of the alkaline phosphatase to 115 U/l (reference value: 35–104 U/l) otherwise with parameters of normal value.

Relying on differential diagnosis an aneurismal bone cyst, giant cell tumor and juvenile bone cyst were discussed.

**Figure 3:**
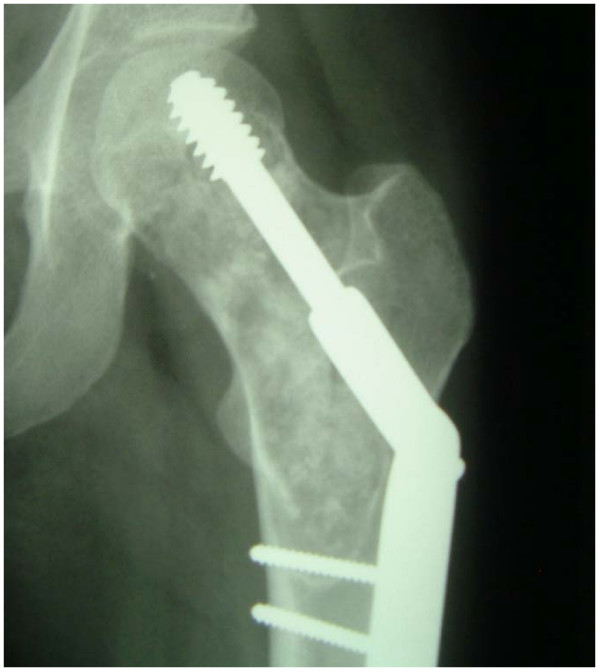
**X-Ray, postoperative: spongiose plastic surgery and internal fixation (DHS)**.

Owing to the pathological fracture, finally the indication of operation seemed sensible and it was carried out by bone biopsy, curettage, spongiose plastic surgery and internal fixation (DHS). (Fig. 3)

Postoperatively the histological examination of the bone material taken intraoperatively made the diagnosis of fibrous dysplasia, which was not taken into consideration in the preoperative considerations during differential diagnosis.

For the assessment of the distribution pattern in conclusion bone scintigraphy was still performed, according to which a monoostotic form of fibrous dysplasia affected the patient.

## Conclusion

Clinically the disease of fibrous dysplasia manifests itself in the form of more or less painful bone deformities, growth disorders and in part extensive osteolyses with transformation zones including spontaneous fracture. Malignant transformations into high-grade-fibro- or osteosarcomas are very rare.

Preferred sites are the long hollow bones, which in some cases are affected in their entirety. Thus the rate of distribution of the lesions run to about 36% affecting the femur, 19% affect the tibia, the ribs and also the calvaria are affected in 10% and 17%, respectively [4].

On X-ray characteristic changes appear, such as the pathognomonic milk glass aspect with reactive peripheral sclerosis, bone expansions, indentations of the inner cortical area and in the femur often the classical shepherd's staff deformity owing to the repetitive microfractures.

By laboratory chemistry the bone metabolism parameter changes in the case of fibrous dysplasia are not necessarily pathognomonic. Nevertheless in the case of the polyostotic form, mostly increases of the alkaline phosphatase (AP) and of the osteocalcin appear. Likewise increased collagen metabolites could be demonstrated (N-Telopeptide).

Furthermore by differential diagnosis secondary osteopathies should be ruled out, e.g. within the scope of hyperparathyroidism.

In the case of fibrous dysplasia the strength of the bone is weaker than normal. Therefore pathological fractures in patients with fibrous dysplasia are observed not infrequently [5,6].

If no fracture or danger of fracture of the bone exists, as a rule conservative therapy is carried out. [7-9]. In most cases, however, therapy by operation is necessary [10,11].

Until about one decade ago the therapy of fibrous dysplasia was largely limited to surgical interventions. Meantime curettage and the filling up of individual foci have gained importance as the definitive choice of treatment, primarily in the case of the monostotic form as well as with smaller symptomatic foci.

In the case of the polyostotic form increasingly a bisphosphonate therapy is used. The first attempt at therapy with the new generation of bisphosphonates was started at the end of the 1980's. Several case studies with Pamidronat showed positive effects exerted on bone density (BMD) and the reduction of pain [12].

In the present case the osteolytic lesion of the proximal femur was categorized as a radiologically benign tumor.

By differential diagnosis on the basis of the MRT finding, an aneurysmal or juvenile bone cyst as well as the diagnosis of a giant cell tumor was given consideration.

Owing to the fracture that has already occurred, correction by operation was done. Histologically the definitive diagnosis of fibrous dysplasia was produced in conclusion.

Fortunately it came to light in the case of the patient within the scope of the follow-up examination that it had to do with a monoostotic form of fibrous dysplasia. In this way the correction was done by surgery in spite of a preoperatively false diagnosis, in accordance with the recommendations of the present literature [13-15].

On the basis of this case report it can be shown that in all cases of an osteolytic lesion it is important to carry out an exact preoperative diagnosis. Furthermore the diagnosis of fibrous dysplasia should be included into the differential diagnosis in order to be able to make with the right decision on therapy in the end.

## Consent

I confirm that *informed written consent *was received for publication of the manuscript.

## Competing interests

All authors certify they not have signed any agreement with a commercial interest related to this study which would in any way limit publication of any and all data generated for the study or to delay publication for any reason. I confirm that all authors have seen and agree with the contents of the manuscript and agree that the work has not been submitted or published elsewhere in whole or in part.

## Authors' contributions

SE performed the clinical and radiologic evaluation of the patient. AW has done the surgery and supervised the evaluation of the patient. SE and AW approved the final manuscript.

## References

[B1] Isefuku S Hatori M Ehara S Hosaka M Ito K Kokubun S Fibrous dysplasia arising from the calcaneus Tohoku J Exp Med 1999 189 227 32 10.1620/tjem.189.227 10674724

[B2] Mirra JM Bone Tumors 1989 Lea & Febiger, Philadelphia 191

[B3] Ozaki T Sugihara M Nakatsuka Y Kawai A Inoue H Polyostotic fibrous dysplasia. A long-term follow up of 8 patients Int Orthop 1996 20 227 32 10.1007/s002640050069 8872545

[B4] Lichtenstein L Jaffe HL Fibrous dysplasia of bone Arch Pathol 1942 33 777 816

[B5] Parekh SG Donthineni-Rao R Ricchetti E Lackman RD Fibrous dysplasia J Am Acad Orthop Surg 2004 12 305 13 15469225 10.5435/00124635-200409000-00005

[B6] Egner S Dobnig H Welkerling H Windhager R Der Stellenwert der Bisphosphonate in der Behandlung der polyostotischen fibrösen Dysplasie Journal für Mineralstoffwechsel 2003 10 2 16 19

[B7] Maldonado I Catalano E Reginato AJ Pathologic fracture of the femoral neck in a female soccer player J Clin Rheumatol 2002 8 1 30 4 10.1097/00124743-200202000-00007 17039197

[B8] Durand S Hamcha H Pannier S Padovani JP Finidori G Glorion C Fibrous dysplasia of the proximal femur in children and teenagers: surgical results in 22 cases Rev Chir Orthop Reparatrice Appar Mot 2007 93 1 17 22 17389820 10.1016/s0035-1040(07)90199-3

[B9] Shih HN Chen YJ Huang TJ Hsu KY Hsu RW Treatment of fibrous dysplasia involving the proximal femur Orthopedics 1998 21 12 1263 6 9867300 10.3928/0147-7447-19981201-06

[B10] Keijser LC Van Tienen TG Schreuder HW Lemmens JA Pruszczynski M Veth RP Fibrous dysplasia of bone: management and outcome of 20 cases J Surg Oncol 2001 76 157 66 10.1002/jso.1028 11276018

[B11] Onoda S Hatori M Yamada N Hosaka M Kokubun S A two-stage surgery for severe femoral neck deformity due to fibrous dysplasia: a case report Ups J Med Sci 2004 109 123 9 15259449 10.3109/2000-1967-102

[B12] Weinstein RS Long-term aminobisphosphonate treatment of fibrous dysplasia: Spectacular increase in bone density J Bone Min Res 1997 8 1314 5 10.1359/jbmr.1997.12.8.1314 9258763

[B13] Chapurlat RD Long-term effects of intravenous pamidronate in fibrous dysplasia of bone J Bone Min Res 1997 12 1746 52 10.1359/jbmr.1997.12.10.1746 9333137

[B14] Stephenson RB London MD Hankin FM Kaufer H Fibrous dysplasia. An analysis of options for treatment J Bone Joint Surg Am 1987 69 400 9 3546323

[B15] Parisi MS Bone mineral density response to long-term bisphosphonate therapy in fibrous dysplasia J Clin Densito 2001 4 167 72 10.1385/JCD:4:2:167 11477309

